# Optimizing solid-based methods for SARS-CoV-2 detection in wastewater: addressing PCR inhibition and variant challenges

**DOI:** 10.1128/aem.00589-25

**Published:** 2025-07-14

**Authors:** Hiroki Ozawa, Kouichi Kitamura, Hiromu Yoshida

**Affiliations:** 1 Microbiological Testing and Research Division, Yokohama City Public Health Research Institute 13946 , Yokohama, Japan; 2 Department of Virology II, National Institute of Infectious Diseases 13511 , Tokyo, Japan; Centers for Disease Control and Prevention, Atlanta, Georgia, USA

**Keywords:** environmental surveillance, wastewater surveillance, SARS-CoV-2, quality control, PCR inhibition

## Abstract

Wastewater surveillance for severe acute respiratory syndrome coronavirus 2 (SARS-CoV-2) RNA is a valuable tool for monitoring coronavirus disease prevalence. However, the dynamics of SARS-CoV-2 and its RNA in wastewater remain largely uncharacterized, leading to uncertainties in viral RNA quantification. This study aimed to improve a recently developed solid-based viral RNA extraction method used in wastewater surveillance programs in Japan. Eighty-four RNA samples extracted from the solid fractions of influent and sludge from two wastewater treatment plants from January to December 2022 were analyzed using reverse transcription quantitative PCR (RT-qPCR) for SARS-CoV-2 and control RNAs. Spiking experiments with a processing control demonstrated that PCR inhibition significantly affected RNA samples from the solid fractions. Contrary to expectations, the commercially available PCR inhibitor removal kit was ineffective, whereas a simple dilution mitigated the inhibitory effect, suggesting the presence of uncharacterized inhibitory factor(s) in the solid fraction. Furthermore, the emergence of the Omicron variant decreased the sensitivity of the CDC N1 RT-qPCR assay. This study demonstrates the improvement of the solid-based method by addressing PCR inhibition and variant emergence and emphasizes the importance of continuous quality control measurements in wastewater surveillance for the effective management of outbreaks caused by evolving viruses, such as SARS-CoV-2.

**IMPORTANCE:**

The standardization of methodologies for SARS-CoV-2 wastewater surveillance has not yet been achieved because of the heterogeneous characteristics of wastewater, even within the same country or sewer system. Studies have demonstrated efficient detection of SARS-CoV-2 RNA in the solid fraction of influent wastewater and sludge. A solid-based methodology has recently been developed for the detection of SARS-CoV-2 RNA; however, there remains potential for further refinement. We assessed the effect of RT-qPCR inhibition in samples from the solid fraction of wastewater using virus-like particles (VLPs) as a processing control. This study emphasizes that the methodology for wastewater surveillance should be regularly evaluated with appropriate quality control (QC) measures and refined based on prevailing circumstances.

## INTRODUCTION

Following the coronavirus disease 2019 (COVID-19) pandemic in 2020, wastewater surveillance for severe acute respiratory syndrome coronavirus 2 (SARS-CoV-2) has become a significant method for complementing other public health surveillance strategies (1). Numerous studies on the detection of SARS-CoV-2 RNA in wastewater have demonstrated an association between sewage RNA concentrations and the number of reported COVID-19 cases. With the end of the Public Health Emergency of International Concern (PHEIC) declaration by the World Health Organization (WHO), the extent of COVID-19 programs has declined, and SARS-CoV-2 wastewater surveillance is gaining importance as a complement to clinical surveillance (1). Environmental surveillance (ES) has been established worldwide to monitor polioviruses as part of polio eradication programs (2–6). Because ES requires technical infrastructure and human resources, existing ES systems can be used to monitor multiple pathogens (7–13). We previously demonstrated the monitoring of enteroviruses and SARS-CoV-2 in wastewater using a polio ES system as research projects (11, 12).

Despite worldwide efforts devoted to the development of detection methods, reliable early detection and precise correlation between SARS-CoV-2 RNA concentrations in sewage and COVID-19 cases remain challenging owing to analytical uncertainty (14). Poliovirus and other enteric viruses are predominantly partitioned into the liquid fraction of wastewater; consequently, this fraction, which is the supernatant obtained from low-speed centrifugation, is used to detect and isolate these viruses. Notably, SARS-CoV-2 RNA was detected in the sewage solid fraction. Solid-based viral RNA extraction methods have recently been developed for the efficient detection of SARS-CoV-2 in wastewater (12, 15–21). Furthermore, it has been reported that sludge in wastewater treatment plants (WWTPs) contains high concentrations of SARS-CoV-2 RNA (22). Nevertheless, the accurate acquisition of viral RNA concentration data remains difficult because of factors, such as virus decay rate, temperature, climatic conditions, differences in the sewer network system, and population size in the catchment area. Among the causal elements of these uncertainties, one laboratory-controllable approach is the mitigation of reverse transcription quantitative PCR (RT-qPCR) inhibition or simply PCR inhibition. Chemical and biological factors in wastewater that can interact with nucleic acids, enzymes, or cofactors may inhibit RT-qPCR; however, they are largely uncharacterized (23), especially for the solid fraction and sludge. PCR inhibitor-removing reagents or inhibitor-tolerant RT-qPCR kits are commercially available, and dilution is easy and cost-effective for assessing and mitigating RT-qPCR inhibition, although it reduces sensitivity.

To minimize uncertainties, including PCR inhibition, wastewater surveillance methods must be evaluated with adequate quality control (QC) (24). Phi6, MS2, and mouse hepatitis virus (MHV) are commonly used as surrogate viruses for evaluating detection methods. The determination of the optimal surrogate particle for use as a processing control presents significant challenges because of the substantial differences in liquid-solid partitioning between spiked materials and indigenous SARS-CoV-2 virions or their RNAs. PMMoV is an indigenous virus used as an internal control (25–27); however, this non-enveloped virus is likely to partition into the liquid fraction (18). We previously demonstrated the utilization of VLP-RNA in spike experiments using environmental samples (28). VLP-RNA is a commercially available non-infectious virus-like particle. The partitioning of VLP-RNA in wastewater may also differ from that of indigenous SARS-CoV-2; however, it can be used to estimate the recovery rate of viral RNA, including the RT-qPCR inhibitory effect.

In the present study, we improved the solid-based viral RNA extraction method for wastewater surveillance in Japan. The effect of RT-qPCR inhibition on solid fractions has not been fully validated and should be assessed using QC protocols to refine the methodology. VLP-RNA was used as a processing control to determine the recovery ratio of the solid-based method. In response to the emergence of new variants of concern, we also tested the detection sensitivity of RT-qPCR primers/probes combined with a PCR inhibition assessment.

## MATERIALS AND METHODS

### Sample collection

Forty-four composite samples of influent wastewater using an automatic sampler (NKS, Japan) (February 2020 and December 2022) and 39 samples of primary settled sludge (July 2020 and December 2022) were collected from both WWTP-A (a combined sewer system) and WWTP-B (separate sewer systems). Samples were collected once or twice a month from the two WWTPs. The combined capacity of the two WWTPs is approximately 1,000,000 inhabitants. All samples were collected in sterile plastic bottles and kept cold during transport to the laboratory. If more than 1 day was required before analysis, the samples were stored at − 80°C.

### RNA purification

The solid-based method has been previously described (12, 18). The influent (250 mL) and sludge (20 mL) samples were centrifuged at 3,000 × *g* for 30  min at 4°C. The resulting sediments were used as the solid fractions. VLP-RNA Extraction Control (10,000 copies/µL) (Meridian Bioscience, Cincinnati, OH, USA) was used as the processing control; VLP-RNA (50 µL) was added to the solid fractions (details in the Quality control subsection). Viral RNA was extracted using the RNeasy PowerSoil Total RNA Kit (Qiagen) according to the manufacturer’s instructions.

### RT-qPCR

SARS-CoV-2 RNA was quantified using the SARS-CoV-2 Direct Detection RT-qPCR Kit (Takara Bio), which is a duplex assay for CDC 2019-nCoV N1 and CDC 2019-nCoV N2 (CDC N1/N2) (29). The Takara One Step PrimeScript III RT-qPCR Mix Kit was used for the single-plex N1 or N2 RT-qPCR assays. The thermal cycling conditions for the RT-qPCR assays were as follows: initial incubation at 52°C for 5 min and initial denaturation at 95°C for 10 s, followed by 45 cycles of denaturation at 95°C for 5 s and primer annealing and extension at 60°C for 30 s for single or duplex CDC N1/N2 assays. VLP-RNA concentrations were determined using the RT-qPCR assay with TaqMan Fast Virus 1-Step Master Mix (Thermo Fisher Scientific), and primers and probe were supplied as VLP Detection Mix of VLP-RNA Extraction Control Red (MDX068, Meridian Bioscience, Cincinnati, OH, USA). The thermal cycling condition was as follows: initial incubation at 52°C for 5 min and initial denaturation at 95°C for 20 s, followed by 45 cycles of 95°C for 10 s and 60°C for 30 s. For the PMMoV assay, RNA samples were diluted 1:100-fold. The RT-qPCR conditions for the PMMoV assay were as previously described (18, 25, 27, 30), and the same thermal cycle as that of SARS-CoV-2 RT-qPCR was used. Thermal cycling was performed using QuantStudio 5 (Thermo Fisher Scientific, Waltham, MA, USA).

### Quality control

PMMoV RNA in the wastewater samples was quantified as an internal control to monitor the substantial loss of viral RNA recovery. In this study, values >10^5^ copies/L in the solid fraction of wastewater were acceptable for PMMoV RNA levels (12, 18). VLP-RNA Extraction Control (10,000 copies/µL) contains an RNA sequence with no homology to any known organism. VLP-RNA (50 µL) was spiked into the solid fraction sample prior to RNA extraction and used to calculate the recovery rate of the solid-based extraction method. RT-qPCR was performed according to the Guidelines for Minimum Information for the Publication of Quantitative Real-Time PCR Experiments (31). All RT-qPCR assays for SARS-CoV-2, PMMoV, and VLP-RNA were performed in duplicate and included both negative and positive standard controls. A 10-fold dilution series of standard RNA controls was prepared to obtain standard curves for each assay. These included 5  ×  10^0^–5  ×  10^3^ gene copies (gc)/reaction (for SARS-CoV-2), 1  ×  10^2^–1  ×  10^5^ (for PMMoV), and 5  ×  10^1^–5  ×  10^4^ (for VLP-RNA). The assay limit of quantification for SARS-CoV-2 was set at 5 gc/reaction as the lowest standard control. This was equivalent to different copy numbers in the influent and sludge samples (400 and 5,000 gc/L, respectively). Reaction mixtures containing fewer than 4 gc/reaction were occasionally amplified in only one duplicate and regarded as negative. To ensure the reliability of the SARS-CoV-2 RT-qPCR assay, AcroMetrix COVID-19 RNA molecular standard (Thermo Fisher Scientific) was used as a reference. A concentration of 50 copies/µL of this reference was tested concurrently to monitor each assay. Ten- or 100-fold dilutions with Tris-EDTA (TE) buffer or OneStep PCR Inhibitor Removal Kits (Zymo Research) were used to eliminate the effects of PCR inhibitory factors. RNA extraction and RT-qPCR were performed in separate laboratory rooms to avoid contamination, and RT-qPCR mixtures were prepared on a clean bench, except for the addition of the template.

### COVID-19 cases

**Fig 1:**
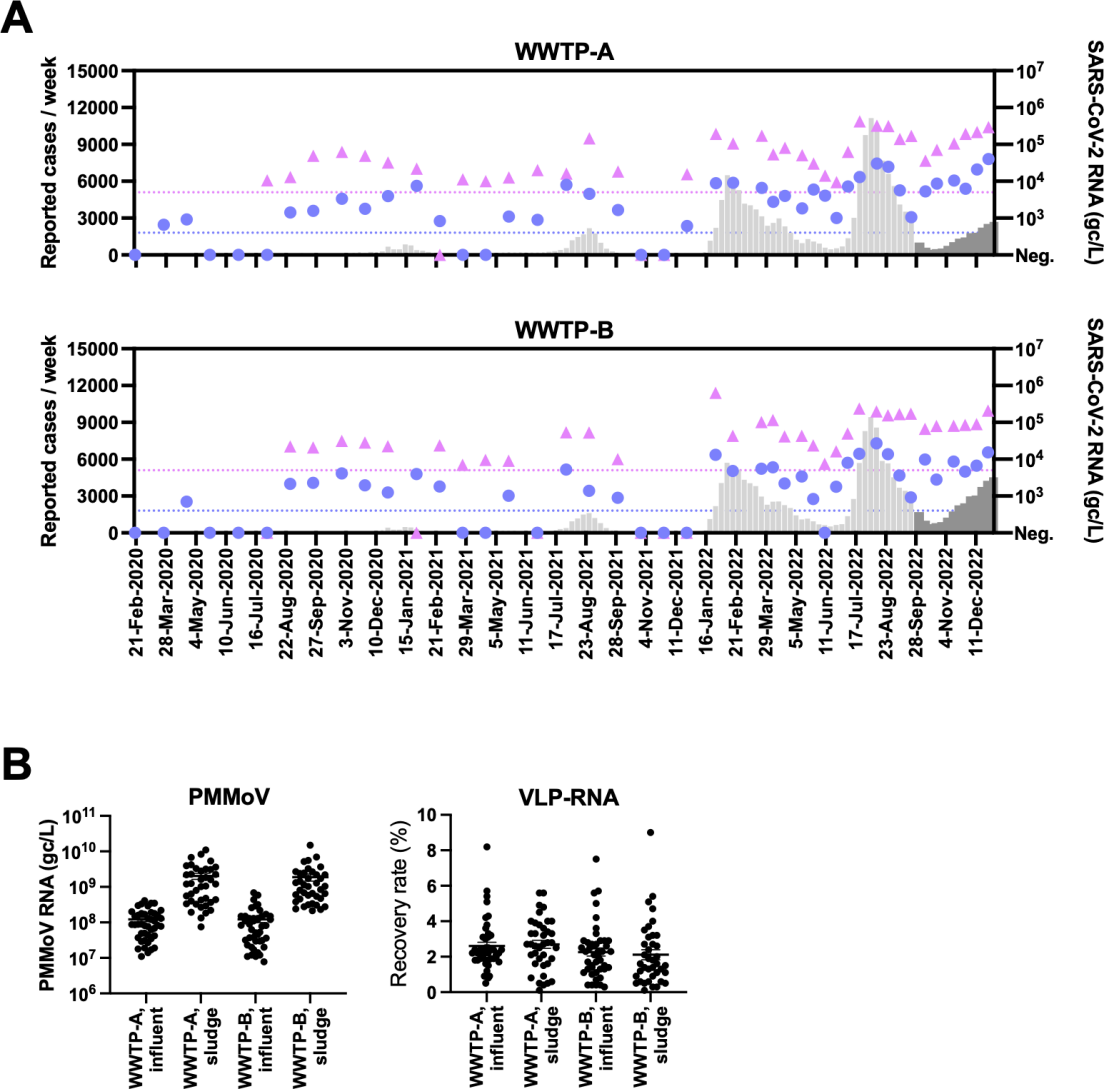
(**A**) Summary of new COVID-19 cases and SARS-CoV-2 RNA concentrations in sewage obtained from WWTP-A and WWTP-B. The number of new COVID-19 cases reported per week in each catchment area is presented (bars). Viral RNA concentrations in influent wastewater (circle) and sludge (triangle) are presented separately. The limits of quantification (LOQs) for wastewater based on RT-qPCR assays were 400 gc/L for influent and 5,000 gc/L for sludge. The reporting system for new COVID-19 cases in Japan was simplified in September 2022 (dark gray bars). (**B**) Scatter plots representing the concentrations of PMMoV RNA (PMMoV) and the recovery rate of VLP-RNA (right) as QC measurements were simultaneously analyzed using SARS-CoV-2 RT-qPCR. The mean and standard error of the mean are presented.

Public data on new COVID-19 cases in administrative districts corresponding to the catchment areas of WWTP-A and WWTP-B were obtained (4 June 2020 to 22 September 2022). On 26 September 2022, the reporting system for new COVID-19 cases in Japan was simplified. New COVID-19 cases were not available at the district level and were estimated based on the population ratio of the district to the municipality (indicated by dark gray bars in Fig. 1 and 5).

### Statistical analyses

Spearman’s rank correlation test was used to analyze the correlation between the sewage SARS-CoV-2 RNA concentrations and the number of new COVID-19 cases. A two-way analysis of variance, followed by multiple comparisons to compare the recovery rates or detected virus copy numbers for each method, was performed using GraphPad Prism 9 (GraphPad Software, San Diego, CA, USA). Statistical significance was set at *P* < 0.05.

## RESULTS

### SARS-CoV-2 RNA detection using a solid-based method from influent and sludge samples

Forty-four composite influent samples (February 2020 to December 2022) and 39 sludge samples (July 2020 to December 2022) were analyzed from WWTP-A and WWTP-B using the solid-based viral RNA extraction method and the CDC N1/N2 duplex RT-qPCR assay. The detection rates of SARS-CoV-2 RNA in the sludge samples were higher than those in the influents (Table 1). Positive signals were detected at approximately the same time as multiple surges of new COVID-19 cases in both WWTPs (Fig. 1). For QC measures, the RNA concentrations of PMMoV and spiked VLP-RNA were quantified as internal and processing controls, respectively. No significant reduction in the PMMoV RNA concentration was observed in any of the tested wastewater samples (Table 1 and Fig. 1B). Recovery rates were calculated from VLP-RNA quantification (average influent, 2.6%; sludge, 2.7% in WWTP-A; influent, 2.3%; sludge, 2.1% in WWTP-B) (Fig. 1B).

**TABLE 1:** Viral RNA detection rates between wastewater influents and sludges

		WWTP-A (combined)		WWTP-B (separate)	
		Composite, influent (*n* = 44)	Sludge (*n* = 39)	Composite, influent (*n* = 44)	Sludge (*n* = 39)
Detection rate	SARS-CoV-2	81.8%	92.3%	72.7%	84.6%
	PMMoV	100%	100%	100%	100%

### Impact of RT-qPCR inhibition on the VLP-RNA recovery rate

**Fig 2:**
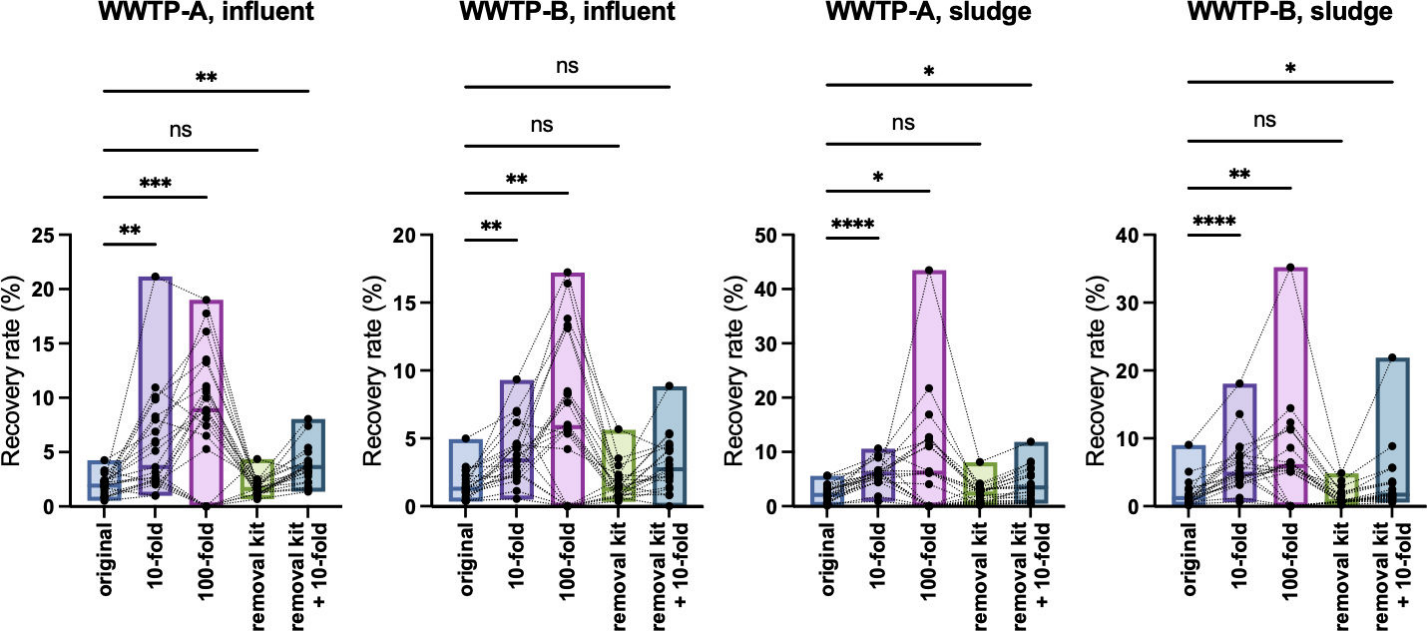
Box plot representing the recovery rate of VLP-RNA. Influent wastewater and sludge samples from WWTP-A and WWTP-B (January–December 2022) were analyzed. To assess the PCR inhibitory effect, 10- or 100-fold dilutions or inhibitor removal kits were used before the VLP-RNA RT-qPCR assay. The median, maximum, and minimum values are presented. The significance of pairwise comparisons was calculated using Friedman’s test with Dunn’s multiple-comparison test. *, *P*  <  0.05; **, *P*  <  0.01; ***, *P*  <  0.005; ****, *P*  <  0.0001. The mean values are presented in Table 2.

Wastewater samples were collected twice a month from January to December 2022. Using RNA samples from this period, the RT-qPCR inhibition effects were assessed by 1:10 or 1:100 dilution with TE, treatment with the PCR inhibitor removal kit, or a combination of 1:10 dilution and the inhibitor removal kit. PMMoV is abundant in wastewater, and 1:10 or 1:100 dilutions are required for RT-qPCR detection. Therefore, only VLP-RNA was used for the RT-qPCR inhibition assessment. The VLP-RNA recovery rates are shown in Fig. 2 and Table 2, indicating an improvement in viral RNA recovery in the 1:10 diluted samples. The 1:100 dilution resulted in a greater increase in the VLP-RNA concentration; however, some samples were undetectable (Fig. 2). Unexpectedly, the PCR inhibitor removal kit was ineffective at improving detection sensitivity in these experiments. The sole effect of dilution was observed in the combination treatment (Fig. 2 and Table 2).

**TABLE 2:** Averages of VLP-RNA recovery rates^*a*^

Recovery rate (%)		Original		10-Fold		100-Fold		Removal kit		Removal kit+ 10-fold	
		Mean	SD	Mean	SD	Mean	SD	Mean	SD	Mean	SD
Influent	WWTP-A	1.97	0.89	5.60	4.74	8.37	5.90	1.72	0.80	3.70	1.70
	WWTP-B	1.60	1.10	3.83	2.14	6.63	5.62	1.62	1.25	3.13	2.01
Sludge	WWTP-A	2.36	1.64	6.02	2.30	8.15	10.3	2.30	1.83	4.12	3.08
	WWTP-B	1.77	2.05	5.99	3.98	6.95	7.84	1.36	1.20	3.44	4.70

^*a*^ SD, standard deviation.

### Selection of primers/probes for SARS-CoV-2 RNA detection during the Omicron surge

**Fig 3:**
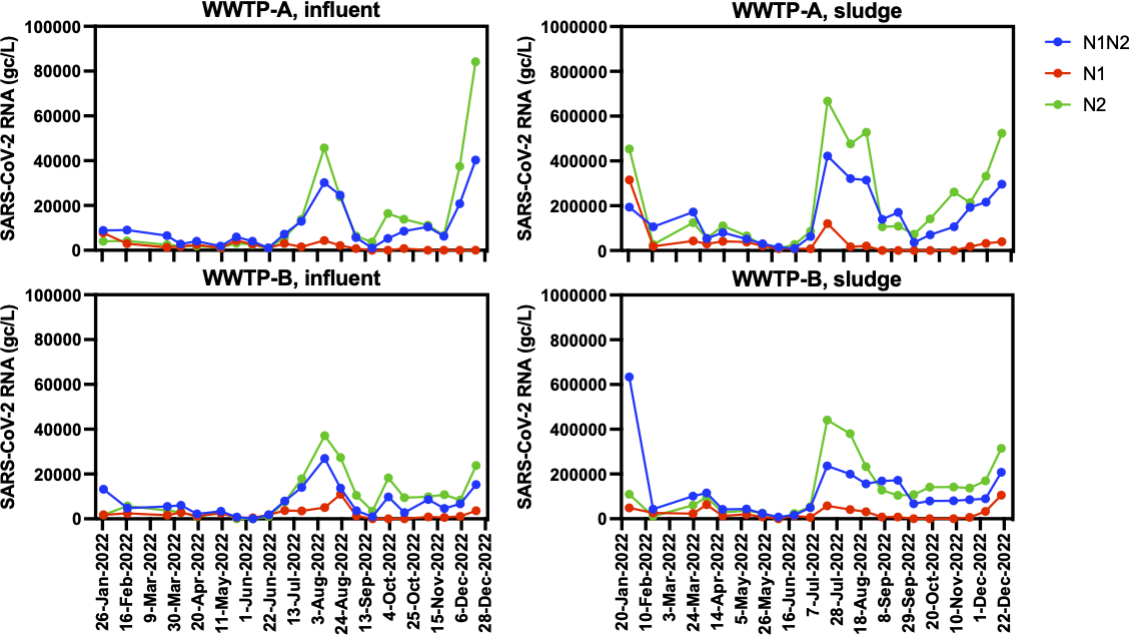
Sewage SARS-CoV-2 RNA concentrations were obtained from influent wastewater and sludge samples from WWTP-A and WWTP-B (January–December 2022). RT-qPCR analyses were performed using CDC N1/N2 duplex (blue), CDC N1 single-plex (red), or CDC N2 single-plex (green).

With the emergence of Omicron variants, a C-to-U mutation appeared at nucleotide position 28311 in the CDC N1 target probe region (32). Furthermore, a subpopulation of the BQ.1* Omicron variant had a secondary C-to-U mutation at nucleotide position 28312 of the N1 probe site (33). Therefore, we assessed the detection sensitivity of CDC N1 and N2 separately in combination with the PCR inhibition assessment. As expected from previous reports, SARS-CoV-2 RNA was detectable using the CDC N1 assay in the first few months of 2022. However, the copy numbers were lower than those for the N2 or N1/N2 results (Fig. 3). After June 2022, the detection rate of the N1 assay decreased substantially. In contrast, the copy numbers of the N2 and N1/N2 assays increased alongside the rise in newly reported cases. The CDC N1 assay failed to detect 1:10 diluted samples across almost all time points because of its reduced sensitivity (data not shown). Hence, we employed the CDC N1/N2 and N2 assays for further analyses.

### Improvement in detection sensitivity after QC measurements

**Fig 4:**
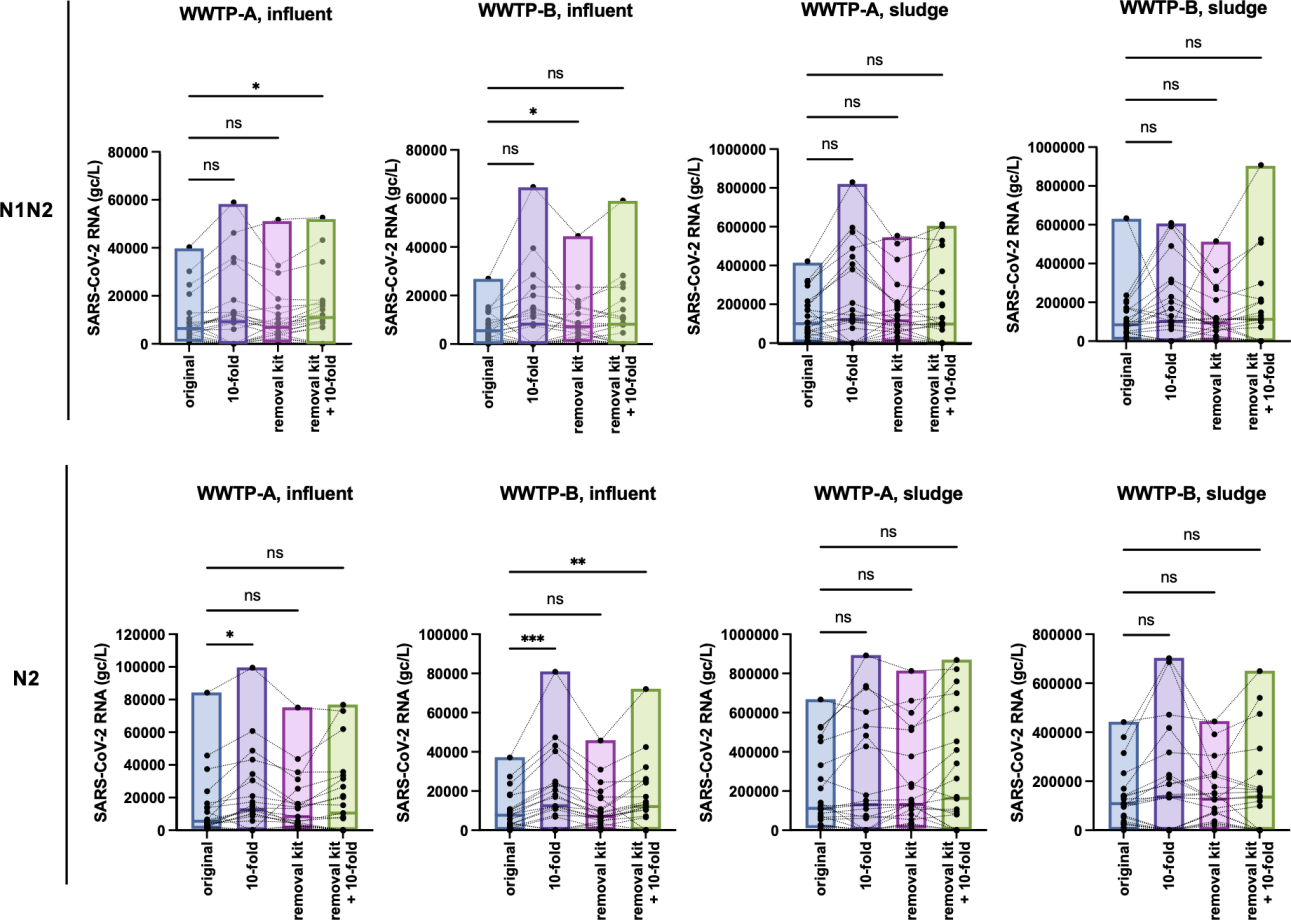
Box plot representing sewage SARS-CoV-2 RNA concentrations (10- or 100-fold dilutions and/or inhibitor removal kits). CDC N1/N2 duplex or N2 single plex. The median, maximum, and minimum values are presented. The significance of pairwise comparisons was calculated using Friedman’s test with Dunn’s multiple-comparison test. *, *P*  <  0.05; ***, *P*  <  0.005.

**Fig 5:**
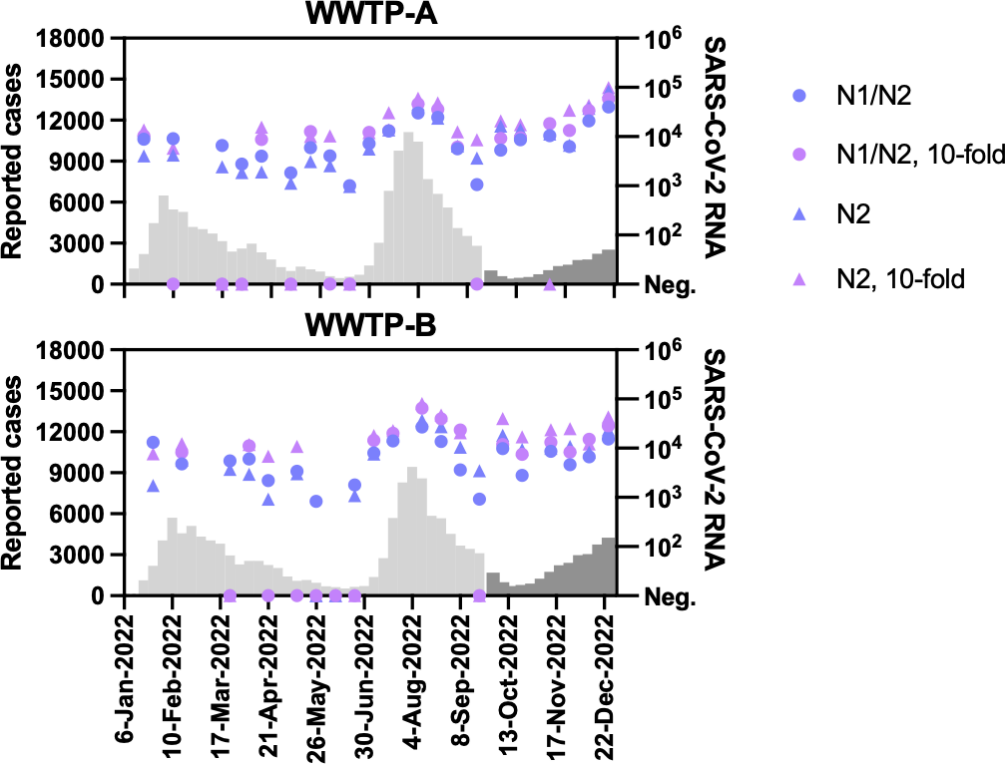
Summary of new COVID-19 cases and SARS-CoV-2 RNA concentrations in influent wastewater samples obtained from WWTP-A and WWTP-B. In addition to the original N1/N2 assay, 10-fold dilution and/or N2 assays were performed. The revised reporting system for new COVID-19 cases (26 September) is presented as gray bars.

Based on the results of the control experiments, we evaluated the RNA samples using RT-qPCR CDC N1/N2 or N2 assays after applying either a 1:10 dilution, PCR inhibitor removal kit, or both (Fig. 4). In the case of SARS-CoV-2 detection, a 1:100 dilution resulted in undetectable levels in almost all samples (data not shown). The results showed a trend toward improved detection sensitivity with dilution and/or inhibitor removal kit. The Friedman test for the paired comparison plot showed a significant difference between the original and 1:10 dilution results with the CDC N2 assay for the WWTP-A and WWTP-B influent samples and the original and the removal kit with the CDC N1/N2 assay for the WWTP-B influent (Fig. 4). Spearman’s test indicated that each analysis showed significant correlations between RNA concentration and the number of reported COVID-19 cases during January–September 2022, except the CDC N1/N2 assay with 1:10 dilution (Table 3). The correlation disappears when including the entire study period from January to December. This suggests that the change in the case reporting system on 26 September may have introduced inconsistencies that affected the analysis (Table 3 and Fig. 5). Taken together, our data indicated that the 1:10 dilution and single-plex CDC N2 assay significantly improved the detection sensitivity of SARS-CoV-2 RT-qPCR. In particular, increases in sensitivity were observed during the latter part of the investigation period (July–December 2022; Fig. 5).

**TABLE 3:** Spearman’s test for correlation between the new COVID-19 cases and the SARS-CoV-2 RNA concentrations

		Jan.–Dec. 2022		Jan.–Sep. 2022	
		Spearman r	*P* value	Spearman r	*P* value
WWTP-A, influent	N1/N2	0.436	0.048	0.696	0.005
	N1/N2, 10-fold dil.	0.212	0.357	0.445	0.098
	N2	0.271	0.234	0.756	0.002
	N2, 10-fold dil.	0.288	0.206	0.534	0.043
WWTP-B, influent	N1/N2	0.599	0.004	0.696	0.005
	N1/N2, 10-fold dil.	0.640	0.002	0.736	0.003
	N2	0.606	0.004	0.851	<0.001
	N2, 10-fold dil.	0.498	0.022	0.717	0.004

## DISCUSSION

An adaptable surveillance system is imperative for effectively managing outbreaks caused by continuously evolving viruses. In the context of the COVID-19 pandemic, notable changes in the prevalence scale and the emergence of novel variants have been observed. Testing capacities have fluctuated, and the strategy for contact tracing has been adjusted in response to evolving situations. Due to uncertainties in viral RNA concentrations in wastewater and variations in RT-qPCR target sequences, it is necessary to employ adaptable detection methods. Continuous revision and updating of methodologies through QC measurements are vital for ensuring accuracy and adaptability.

In this study, VLP-RNA was used to control the processing of the solid-based method. As mentioned above, neither indigenous viruses nor spike materials specific to the solid fraction were available since most of the spiked materials are partitioned into liquid fractions. To overcome this limitation, VLP-RNA was added to the solid fraction after centrifugation. Nevertheless, we used this material only to confirm the recovery rate and not for the normalization of SARS-CoV-2 RNA concentrations. It is well known that fecal and soil samples contain PCR inhibitors, such as humic acids, heme, polysaccharides, polyphenols, fluvic acids, lipids, and dyes (23). To mitigate the effects of these inhibitory factors, PCR inhibitor removal kits and inhibitor-resistant RT-qPCR reagents are commercially available. However, our VLP-RNA experiments suggested that they were ineffective, whereas a 1:10 dilution mitigated PCR inhibition, indicating the presence of unknown inhibitors in the solid fraction of wastewater. The results from the 1:100 dilution showed higher sensitivity in the VLP-RNA experiment (Fig. 2), but the concentration of SARS-CoV-2 RNA was too low to be diluted to 1:100. The inhibitor levels and diversity in wastewater may vary depending on the sampling sites, and perhaps even the sampling timing. These are influenced by the population size, industry, and agriculture in the catchment area of the WWTP. Further investigation is required to identify the unknown inhibitors in wastewater.

A subpopulation of the Omicron variants BA.5.2, BF.5, and BQ.1.1 has two mutations within the nucleotide sequence corresponding to the CDC N1 probe site (C28311T and A28330G in BA.5.2 and BF.5, C28311T and C28312T in BQ.1.1) (32, 34, 35). These mutants tended to dominate from mid-2022 until the end of the year. Previous studies have demonstrated that these mutations do not affect the detection sensitivity of SARS-CoV-2 RT-qPCR in clinical samples; however, they cause underestimation of the virus concentration or pseudo-negative results in wastewater surveillance (33, 36). Our results also showed a substantial decrease in the SARS-CoV-2 RNA concentration in the N1 assay compared to the N1/N2 and N2 assays during July–December 2022, whereas comparable levels of viral RNA were detected in each assay, including N1 in the January 2022 sample (Fig. 3). An underestimation of the viral RNA concentration in the N1 assay was also demonstrated in a previous study (35). These data suggest that multiplex RT-qPCR assays, such as the CDC N1/N2 assay, are advantageous for mitigating the risks of single-plex assays against variable virus sequences. Although the detection sensitivity improved with these changes, the correlation between COVID-19 cases and sewage viral RNA concentrations did not always improve (Fig. 5 and Table 3). To clarify the relationship between the number of reported cases and sewage RNA concentrations, it is necessary to use mathematical models rather than simple correlations to quantitatively analyze how multiple factors interact and influence each other.

In summary, we implemented long-term SARS-CoV-2 RNA wastewater surveillance using QC measurements. Using a processing control VLP-RNA, the impact of PCR inhibitors was comprehensively analyzed, and an effective mitigation method was identified. The sensitivity of the RT-qPCR primer/probe sets was also evaluated during the surveillance period, and the sensitivity of the mismatched probe was demonstrated. Thus, the solid-based method for detecting sewage SARS-CoV-2 has been improved. This study emphasizes the importance of QC measurements for long-term ES, especially for emerging and evolving viruses such as SARS-CoV-2.

## AFTER EPUB

*[This article was published on 14 July 2025 with an error in the Fig. 3 caption. The caption was corrected in the current version, posted on 29 July 2025.]*
