# Erratum for Ozawa et al., “Optimizing solid-based methods for SARS-CoV-2 detection in wastewater: addressing PCR inhibition and variant challenges”

**DOI:** 10.1128/aem.01009-26

**Published:** 2026-06-04

**Authors:** Hiroki Ozawa, Kouichi Kitamura, Hiromu Yoshida

## ERRATUM

Volume 91, no. 8, e00589-25, 2025, https://doi.org/10.1128/aem.00589-25. Author affiliation 1 should read “Microbiological Testing and Research Division, Yokohama City Institute of Public Health, Yokohama, Japan.”

